# Politically Contaminated Clothes, Chocolates, and Charities: Distancing From Neutral Products Liked by Out-Group or In-Group Partisans

**DOI:** 10.1177/01461672241298390

**Published:** 2024-11-20

**Authors:** Arvid Erlandsson, Artur Nilsson, Jennifer Rosander, Rebecka Persson, Leaf Van Boven

**Affiliations:** 1 Linköping University, Sweden; 2 University of Bergen, Norway; 3 Stockholm School of Economics, Sweden; 4 University of Colorado, Boulder, USA

**Keywords:** affective political polarization, social distancing, party over policy effect, political boycotting, social identity signaling

## Abstract

This research demonstrates that people distance themselves not just from out-group partisans or policies but also from completely neutral and apolitical consumer products that have been “contaminated” simply by being preferred by the political out-group. Using large representative samples of Swedish adults, we investigated how aesthetic judgments of clothes (Study 1), evaluations of chocolate bars (Study 2), and allocations to charitable organizations (Study 3) were influenced by a randomly assigned association between these products and the leader or supporters of the participant’s least- or most-liked political party. Products liked by the least-liked party became less attractive in all studies; the results were mixed for products liked by the most-liked party. Study 4 found that the presence of in-group-observers increased distancing from products liked by the least-liked party, indicating that self-presentational concerns bolster political distancing. These results suggest that affective political polarization influences our lives more subtly and profoundly than previously known.

Affective political polarization is the human tendency to dislike and distrust the political out-group while liking and trusting the political in-group (Druckman & Levendusky, 2019; Iyengar et al., 2019). In this article, we investigate a subtle and yet unexplored manifestation of affective polarization that occurs when people’s evaluations of neutral and apolitical products change when the products become indirectly associated with partisan individuals. For instance, did cherry-vanilla ice cream taste worse for liberals, but better for conservatives after it was revealed that it is Donald Trump’s favorite flavor (US Weekly, 2015)?

In four studies conducted using large representative samples in Sweden, we investigate both out-group- and in-group-based manifestations of this kind of indirect political distancing in the aesthetic, gastronomic, and prosocial domains. Specifically, we test if people evaluate clothes as less (or more) beautiful, chocolates as less (or more) appetizing, and charities as less (or more) worth donating money to, after learning that leaders or supporters of their least favorite—or favorite—political party like or dislike these neutral products. We also investigate social identity signaling as a moderator to this phenomenon by testing if the effects are stronger with publicly expressed preferences.

## Background

It might seem strange to avoid (or approach) a neutral product such as an ice cream flavor, just because political opponents or allies favorably evaluated it. Still, it is plausible that people make politically motivated judgments even in apolitical domains as a way of upholding cognitive consistency (Simon & Holyoak, 2002), or to signal their identity to others (Brown, 2000). Social identities can profoundly affect cognition, affect, and behavior, such as food preferences (Hackel et al., 2018) and olfactory judgments (Coppin et al., 2016), and partisan identities are some of the strongest social identities in modern post-industrial democracies (Iyengar et al., 2012), and can be positive (e.g., identification with the most-liked party) or negative (e.g., identification as an opponent of the least-liked party; Medeiros & Noël, 2014).

*Affective polarization* manifests as animosity between political in- and out-groups and is high in the United States (Druckman et al., 2021; Iyengar et al., 2019) and other countries (Flores et al., 2022; Reiljan, 2020) including Sweden (Reiljan & Ryan, 2021; Renström et al., 2021). Longitudinal studies suggest that negative feelings toward political out-groups grew more intense in the United States from 2010 to 2020 (Finkel et al., 2020), and expressions of affective polarization can be seen as socially desirable (Connors, 2023). Measures of affective polarization can be divided into two broad categories (Druckman & Levendusky, 2019). One entails measures of self-rated emotions or trust toward political in-groups and out-groups (Lelkes & Westwood, 2017; Levendusky, 2013). The other includes preferred social distance measured in terms of questions about, for instance, how comfortable people say they would be to work with an out-group-partisan (vs. in-group-partisan) or to have their child marry a person from the political out-group (Klar et al., 2018).

Affective polarization can manifest in various behaviors. *Direct political distancing* occurs when people avoid contact with individuals or groups that are explicitly partisan, for example, when deciding whom to date (Mallinas et al., 2018), where to live (Motyl et al., 2014), whom to hire for a job (Gift & Gift, 2015), and how much time to spend at a Thanksgiving dinner (Chen & Rohla, 2018). Direct political distancing exacerbates political polarization but could serve as a useful behavioral heuristic insofar as avoiding contact with an out-group may result in less intergroup hostility (Gift & Gift, 2015).

*Indirect political distancing* occurs when a potentially neutral idea or object is “contaminated” simply by being indirectly associated with an individual from a political out-group. For example, people tend to dismiss arguments or suggestions made by their political out-group (Frimer et al., 2017) and to evaluate policies proposed by their political out-group as unattractive and policies suggested by the political in-group as attractive—the so-called party over policy-effect (Cohen, 2003; Verkuyten & Maliepaard, 2013). Furthermore, perceived partisanship can influence consumer behaviors such as boycotting and buycotting (Kam & Deichert, 2020). People are less likely to purchase discounted gift cards when these have been used in a political campaign of their out-group (vs. in-group) party (McConnell et al., 2018), less likely to eat at a restaurant that has sponsored their political out-group (Panagopoulos et al., 2020), and less likely to recommend music and poems performed by out-group artists (Pomerance & Van Boven, 2025).

A general methodological problem in research on indirect distancing is how directional reasoning can be separated from other kinds of non-directional motives (Tappin et al., 2020). In the party over policy-paradigm, people might use the partisanship of the source as an informational cue about what they would think about a complex issue if they were fully informed, for instance, when evaluating complex policies about the climate (Cole et al., 2022) or COVID-19 (Flores et al., 2022). Similarly, in most previous studies on political consumerism, the products or services were either inherently political or directly affected political parties or partisans.

To control for the potentially accuracy-driven and justifiable motives people might have for avoiding socialization with out-group partisans, dismissing policies suggested by the out-group, and avoiding brands that sponsor the out-group, we set out to investigate if attitudes toward completely apolitical products are affected simply by information about what the political out-group and in-group think about these products. Following the suggestion by Van Boven and Sherman (2021) to separate in-group and out-group influences on political behavior, we hypothesized that two effects would occur:

**Hypothesis 1 (H1):** A negative *out-group effect*: People will evaluate products more negatively after these have been associated with their least-liked party.
**Hypothesis 2 (H2):** A positive *in-group effect*: People will evaluate products more positively after these have been associated with their most-liked party.

Theoretically, these effects can be explained by a strive for balance (Heider, 1946), dissonance reduction (Festinger, 1957), or general cognitive consistency (Simon & Holyoak, 2002). Learning that otherwise desirable consumer products are preferred by a disliked out-group can make one’s set of held attitudes and preferences imbalanced, and this can evoke psychological processes intended to restore consistency. Because it is easier to change attitudes toward clothes, chocolates, and charities than it is to change attitudes toward political parties, people might revise their product preferences to restore cognitive consistency.

Moreover, studies suggest that expressions of affective polarization can provide reputational benefits and may be driven by self-presentation motives (Connors, 2023; Moore et al., 2023). Consequently, we predict (in Study 4) that identity signaling will influence the distancing effect, such that preferences for politically contaminated products will differ based on whether they are expressed privately or publicly in front of in-group members (Berger & Heath, 2008). This predicted pattern aligns with classic research showing that self-presentation amplifies cognitive dissonance effects (Baumeister & Tice, 1984; Paulhus, 1982), indicating that self-presentational concerns could heighten the tendency to distance oneself from products associated with political out-groups.

Furthermore, we investigate whether in-group and out-group effects are equally strong for leftist and rightist participants (i.e., ideologically symmetrical) or more pronounced among leftists or rightists (i.e., ideologically asymmetrical). Many studies have found that United States conservatives (vs. liberals) exhibit greater in-group centrism, relational motivation, and perceived in-group consensus (Jost et al., 2018; Waytz et al., 2019) and more proneness to cognitive biases and motivated thinking (Baron & Jost, 2019). Other studies have shown similarities in politically motivated cognition and in-group bias across the political spectrum (Crawford & Brandt, 2020; Ditto et al., 2019), and some recent studies have even suggested that the tendencies to dislike political opponents and empathize less with them is stronger among leftists than rightists (Casey et al., 2025; Ridge et al., 2024).

A significant feature of the current research is that all data were collected from nationally representative samples of Swedes. Most previous studies have used samples from the United States, which has a long-standing biparty system that is far from representative of the rest of the world and may exacerbate bipolar attitude polarization. There is a need for research with representative samples from other countries (Gidron et al., 2019), particularly those with multi-party systems and multifaceted ideological landscapes (Boxell et al., 2020; Knudsen, 2021). Whereas opposing the Republican party implies supporting the Democrat party in the United States, there are several possible most- and least-liked party combinations in multi-party systems. At the time of these studies, there were eight parties in the Swedish parliament.^1^ The Left, Green, Social Democratic, and Center parties, which subscribe to socialist, environmentalist, social democratic, and socio-liberal ideologies, respectively, currently make up the left-wing block. The Liberal, Christian Democratic, Moderate, and Sweden Democratic parties, which subscribe to liberal, social-conservative, liberal-conservative, and nationalist-conservative ideologies, respectively, currently represent the right-wing block (see Table 1).^2^

**Table 1.** Information About Participants in Each Study.

Information	Study 1: Clothes (March 2021)	Study 2: Chocolates (March 2022)	Study 3: Charities (March 2022)	Study 4: Charities, Chocolates, Fruits (March 2023)
**Valid N [excluded due to missed attention check]**	638 [57]	813 [174]	1,239 [151]	1,295 [324]
**Female/Male/ Unclassified**	51.3 / 48.4 / 0.3 %	50.6 / 49.1 / 0.4%	50.2 / 49.4 / 0.4%	51.2 / 48.4 / 0.2 %
**Mean age (*SD*)**	49.31 (16.26) years	48.72 (16.21) years	48.34 (16.48) years	≈45–50 years (age measured in ranges)
**Highest completed education**	NA	4.4% Elementary or less 36.5% High school 12.5% University/ college courses 46.5% University/ college degree	5.2% Elementary or less 37.4% High school 13.8% University/ college courses 43.6% University/ college degree	5.8% Elementary or less 37.5% High school 12.3% University/ college courses 44.2% University/ college degree
**Place of living**	NA	25.2% Countryside 19.7% Small town 27.4% Small city 27.7% Large city	22.9% Countryside 18.7% Small town 26.6% Small city 29.7% Large city	NA
**Most-liked party %** **[Least-liked party %]**				
***Leftist block***				
Left (1.80–2.36)^a^	11.6% [10.8%]	9.6% [10.3%]	11.2% [11.5%]	9.9% [10.6%]
Social democratic (2.84–3.16)^a^	26.2% [6.3%]	28.2% [2.2%]	28.8% [3.3%]	32.3% [3.0%]
Green (2.40–3.35)^a^	6.7% [23.4%]	5.0% [28.3%]	3.8% [27.0%]	5.1% [26.6%]
Center (3.56–4.08)^a^	9.4% [2.5%]^b^	8.9% [3.1%]	7.7% [2.5%]	6.9% [2.1%]
***Rightist block***				
Liberal (4.46–4.97)^a^	4.2% [2.5%]^b^	4.6% [1.2%]	4.0% [1.9%]	6.1% [0.8%]
Christian democratic(4.95–5.80)^a^	5.5% [3.4%]	4.3% [3.7%]	6.1% [4.0%]	4.3% [4.2%]
Moderate (5.39–5.70)^a^	21.8% [1.7%]	24.4% [2.1%]	22.9% [2.7%]	19.5% [2.2%]
Sweden democratic (5.09–5.58)^a^	14.6% [49.4%]	15.1% [49.1%]	15.5% [47.0%]	15.9% [50.4%]

a Number ranges in parentheses indicate the 95% confidence interval for means on political self-placement (1 = *extremely leftist*; 7 = *extremely rightist*), based on the party participants chose as their most liked. This self-placement data was collected in an unrelated study in early 2024 (*N* = 401). A similar pattern was found in Study 1 (conducted in early 2021), except that the Center and Liberal parties were closer to each other at that time. ^b^ The Center and Liberal parties were not classified as Leftist or Rightist in Study 1 (see note 2).

## The Current Research

Four experiments tested whether people’s attitudes and preferences regarding otherwise neutral products are affected by whether the products have been associated with the most- or least-liked political party. Study 1 investigated this in the aesthetic domain by testing if people perceive clothes as less [more] beautiful and valuable after learning that identical clothes are worn by leaders of their political out-group [in-group]. Study 2 manipulated descriptive social norms, investigating how knowledge about chocolate preferences among supporters of political out-groups and in-groups influences participants’ evaluations of these chocolates. These studies used a repeated-measures design, in which participants evaluated the same products before and after an arbitrary positive or negative association between these products and the participants’ most- or least-liked party was introduced. Study 3 manipulated descriptive norms through a between-group design, investigating how information about the preferences of out-group and in-group supporters influenced real financial allocation decisions in the charitable domain. Finally, Study 4 investigated if the presence (vs. absence) of in-group observers would exacerbate the tendency to avoid or approach politically associated neutral products.

In all studies, we classified participants as leftists or rightists based on their most-liked party. We repeated key analyses with political placement based on the least-liked party, based on only clear partisans (i.e., consistently classified as leftists or rightists both in terms of most- and least-liked party). Results of these robustness tests are reported in each study’s Supplementary Material.

## Open Practices Statement for All Studies

All studies were administered through Qualtrics, and samples that were representative of the adult Swedish population in terms of gender, age, and political affiliation were recruited through the independent data collection company PFM Research. We report preregistered sample sizes, all manipulations, and all measures for all studies (we do not report results for every measure, but all measures are included in the openly accessible raw data files). Sample sizes were determined based on a priori power analyses and the availability of resources. Participants in all studies were excluded only if they failed the embedded attention check (see Table 1). As explained below, we deviated some from the preregistered plan for the analyses. All stimuli material, Supplementary Material, preregistrations, raw data, variable codebooks, and analysis code can be found the OSF-page for each study.

## Study 1: Politically Contaminated Clothes

Participants evaluated clothes before and after these clothes were associated with political parties. The associations were induced by showing pictures of famous politicians wearing the clothes. The participants also rated clothes worn by non-politicians. Unlike previous research on boycotting (e.g., McConnell et al., 2018; Panagopoulos et al., 2020), the partisan association with the products was indirect—the clothing brands were not sponsoring any of the politicians, and there was no political or personal gain or loss for the politician if people would change their aesthetic judgments. While research has shown that people avoid wearing clothes that have been worn by ill-fated or immoral people (Rozin et al., 1994), our study asked participants to evaluate clothes that are identical to those worn by politicians. Thus, it investigated a more indirect, symbolic, and arguably more common type of distancing that does not involve physical contact.

### Method

We requested 600 participants, and a sensitivity power analysis showed that this would give us 80% power to detect an effect size of *d* = .10 in paired *t*-tests of changes in evaluations of clothes. The final sample size was 638 (see Table 1). Participants were told that the purpose of the study was to investigate how people perceive and evaluate clothes.

The participants were first shown 17 pictures depicting pieces of formal clothing (e.g., a dress, jacket, blouse, or blazer) in a randomized order. The clothes were always worn by a person, but the pictures were cropped so that the face of the wearer was not visible. For each picture, the participants evaluated the “design and fit” and “color” of the depicted clothes on visual analog scales ranging from −50 (“Very ugly”) to +50 (“Very beautiful”). They also rated their willingness to purchase clothes that were identical to those shown in the pictures for themselves or for someone they knew by choosing the highest price range they would be willing to pay. The suggested ranges were in 250SEK (≈ $30) increments so that a score of 1 represented 0–250 SEK and a score of 8 represented 1751–2000 SEK (9 = 2001 SEK or more). For brevity, we report results based on the aggregate of these variables in the text,^3^ but results for each dependent variable are reported in Table 2.

**Table 2.** Mean Change Scores (and Standard Deviations) and Cohen’s d [95% CI] Derived From Paired t-Tests for All Participants, Only Leftist Participants, and Only Rightist Participants for Each Dependent Variable. Positive [Negative] Change Scores Indicate That Revealing the Identity of the Wearer Made Evaluations More Positive [Negative].

	Change scores			
	All participants *n* = 638	Only leftists *n* = 284	Only rightists *n* = 267	Test of difference of leftists and rightists change scores.
**Least-liked party clothes**				
Design	−3.70 (14.45) −.26 [−34, -.18]	−2.92 (13.78) −.21 [−.33, −.09]	−4.85 (15.53) −.31 [−.44, −.19]	*t*(549) = −1.54, *p* = .123, *d* = −.13 [−.30, .04]
Color	−3.70 (14.52) −.26 [−33, −.18]	−2.56 (15.07) −.17 [−.29, −.05]	−5.52 (14.48) −.38 [−.51, −.26]	*t*(549) = −2.34, *p* = .019, *d* = −.20 [−.37, −.03]
Willingness to pay	−0.06 (0.95) −.07 [−15, .01]	−0.04 (0.95) −.04 [−.16, .07]	−0.11 (0.97) −.11 [−.23, .00]	*t*(549) = −0.86, *p* = .392, *d* = −.07 [−.24, .09]
**Most-liked party clothes**				
Design	+2.74 (12.87) .21 [13, .29]	+1.77 (11.21) .15 [.03, .27]	+4.47 (13.74) .33 [.20, .45]	*t*(549) = 2.49, *p* = .014, *d* = .21 [.05, .38]
Color	+0.41 (12.38) .03 [−05, .11]	−0.90 (11.50) −.08 [−.20, .04]	+2.12 (13.22) .16 [.04, .28]	*t*(549) = 2.87, *p* = .005, *d* = .24 [.08, .41]
Willingness to pay	+0.20 (0.91) .23 [15, .30]	+0.06 (0.74) .08 [−.03, .20]	+0.38 (1.04) .37 [.25, .49]	*t*(549) = 4.21, *p* < .001, *d* = .36 [.19, .53]
**Non-political clothes**				
Design	+0.62 (9.15) .07 [−01, .15]	+0.09 (9.56) .01 [−.11, .13]	+1.27 (9.29) .14 [.02, .26]	*t*(549) = 1.46, *p* = .145, *d* = .13 [−.04, .29]
Color	+0.52 (9.25) .06 [−02, .13]	+0.23 (9.77) .02 [−.09, .14]	+0.86 (8.82) .10 [−.02, .22]	*t*(549) = 0.79, *p* = .433, *d* = .07 [−.10, .23]
Willingness to pay	+0.10 (0.63) .16 [.08, .24]	+0.07 (0.60) .13 [.01, .24]	+0.15 (0.66) .23 [.11, .35]	*t*(549) = 1.39, *p* = .165, *d* = .12 [−.05, .29]

On the next page, participants reported current age, gender identity, fashion interest (1 = “No interest,” 7 = “Very interested”), and feelings toward each of the eight parties in the Swedish parliament (−50 = “Strongly dislike,” +50 = “Strongly like”). They also chose the political parties they liked the most and the least and reported their left-right self-placement (−50 = “Strongly leftist,” +50 = “Strongly rightist”).

They thereafter evaluated the same 17 clothes (in a randomized order) as in the first part of the study as well as two additional pictures of clothes that were presented before the others.^4^ Like at Time 1, they evaluated design, color, and how much they were willing to pay for identical clothes, using the same scales. The only difference was that the pictures were no longer cropped, which means that the face of the wearer was visible at Time 2.

Three of the reoccurring pictures were stock photos depicting unknown non-politicians (one man and two women); the other 14 pictures showed well-known Swedish politicians. We included one male and one female politician from each of the three left-wing parties (Left, Green, and Social Democrat) and from each of the three right-wing parties (Christian Democrat, Moderate, and Sweden Democrat). Because the Center and Liberal parties were not unequivocally left- or right-wing at the time of data collection, they were represented by just one picture each. We used existing photographs of politicians, but we matched pairs of pictures for the left- and right-wing parties so that the age, gender, pose, and type of clothing were as similar as possible. The name and party affiliation of the politician were written above the pictures to increase party salience. Finally, participants again reported their feelings toward each of the eight political parties using the same response scale as before.

### Results

**Figure 1.**
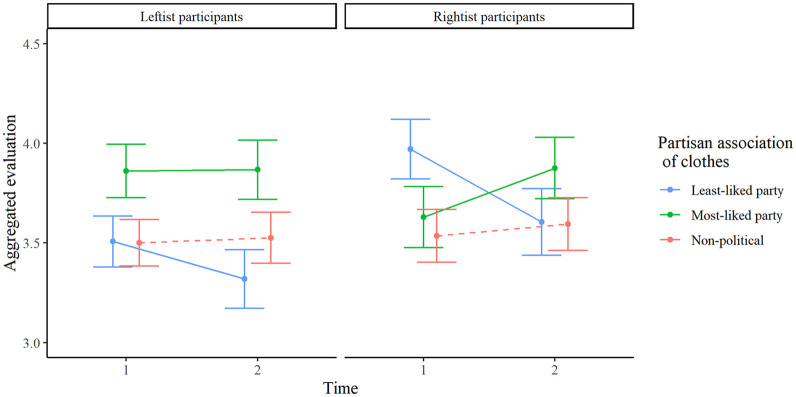
Changes in Aggregated Evaluations From Time 1 to Time 2 for When the Clothes Were Worn by Politicians From the Participants’ Least-Liked Party (Blue Line), Most-Liked Party (Green Line), and by Non-Politicians (Red Dotted Line). A Downward Blue Slope Indicates a Negative Out-group Effect. An Upward Green Slope Indicates a Positive In-group Effect. Error Bars Represent 95% Confidence Intervals.

Participants who chose the Left, Green, or Social Democratic party as their most-liked party (*n* = 284) were classified as leftists, whereas those who chose the Christian Democratic, Moderate, or Sweden Democratic party were classified as rightists (*n* = 267). Their aggregate evaluations of clothes associated with the most- and least-liked parties, and with non-politicians, are illustrated in Figure 1. Mean change scores (Time 2 minus Time 1) for each dependent variable are reported in Table 2.

An initial omnibus 3 (Partisan association of Clothes: Most-liked party/Least-liked party/Non-political) × 2 (Time: 1[before partisan association]/2[after partisan association]) × 2 (Participant partisanship: leftist/rightist) mixed ANOVA with aggregate evaluation as the dependent variable rendered a significant Clothes × Time interaction effect, *F*(2, 548) = 40.74, *p* < .001, η_p_^2^ = .13, implying that evaluations of clothes were influenced by the induced partisan association. The three-way interaction was also significant *F*(2, 548) = 9.80, *p* < .001, η_p_^2^ = .04, suggesting that leftists and rightist participants were differently influenced by the induced partisan associations. We replicated the analysis with mixed linear modeling, which yielded similar results (see OSF).^5^

We followed this up by investigating each type of clothing separately. A 2(Time) × 2(Participant partisanship) ANOVA with general evaluations of clothes worn by *least-liked party* politicians as the dependent variable revealed a *negative* main effect of Time, *F*(1, 549) = 39.70, *p* < .001, η_p_^2^ = .07, implying that clothes associated with participants’ least-liked party were evaluated less positively after the identities of the wearers had been revealed. This supports our *out-group hypothesis*. There was also a main effect of partisanship *F*(1, 549) = 12.51, *p* < .001, η_p_^2^ = .02, such that rightists rated the clothes as more attractive overall. The interaction was marginally significant, *F*(1, 549) = 4.18, *p* = .041, η_p_^2^ = .01, indicating a stronger out-group effect among rightists (*d* = -.34 [-.48, -.22]) than among leftists (*d* = -.19 [-.31, -.07]). This interaction effect remained significant when we entered changes in the aggregate evaluation of non-political clothes (Time 2 minus Time 1) and fashion interest as covariates, *F*(1,547) = 5.60, *p* =.018, η_p_^2^ = .01, but not when we used alternative ways to classify participants as leftists or rightists (see Supplementary Material).

A similar 2*2 ANOVA with evaluations of clothes worn by *most-liked party* politicians as the dependent variable revealed a *positive* main effect of Time *F*(1, 549) = 29.51, *p* < .001, η_p_^2^ = .05, implying that clothes associated with the most-liked party were evaluated more positively. This supports our *in-group hypothesis*. There was no main effect of participant partisanship, *F*(1, 549) = 1.42, *p* = .234, η_p_^2^ < .01, but the interaction effect was significant, *F*(1,549) = 16.54, *p* < .001, η_p_^2^ = .03; there was a positive in-group effect among rightists (*d* = .36 [.23, .48]) but not leftists (*d* = .07 [−.05, .19]). This interaction remained significant adjusting for aggregate evaluation of non-political clothes and fashion interest, *F*(1,547) = 13.84, *p* <.001, η_p_^2^ = .02, and when using any of the alternative ways to classify participants as leftists or rightists.

For clothes worn by non-politicians, there was a positive main effect of Time *F*(1, 549) = 10.69, *p* = .001, η_p_^2^ = .02, indicating that the clothes were rated more positively at Time 2. However, this increase was smaller than the increase for clothes worn by most-liked party politicians, *t*(637) = 2.84, *p* = .005, *d* = .11. There was no main effect of participant partisanship, *F*(1, 549) = 0.13, *p* = .715, η_p_^2^ < .01, and the interaction effect was not significant *F*(1, 549) = 2.85, *p* = .092, η_p_^2^ = .01.

### Summary of Study 1

Study 1 found support for both the out-group- and in-group-effects: Participants evaluated clothes identical to those worn by leaders of their least-liked political party as less attractive and clothes identical to those worn by leaders of their most-liked party as more attractive. These results demonstrate that aesthetic evaluations of neutral apolitical clothes can be contaminated by irrelevant political associations. The results also revealed ideological asymmetries in that particularly the positive in-group effect was more pronounced among rightists.

## Study 2: Politically Contaminated Chocolates

Study 2 replicated and extended the results of Study 1. It used a similar experimental paradigm, wherein participants evaluated neutral products before and after a partisan association was introduced, but it used chocolate bars rather than clothes as the neutral product. This creates a more conservative test of our hypothesis, as chocolates are inherently less identity-relevant than clothes (Feinberg et al., 1992). Another difference is that Study 2 used descriptive party norms (people who vote for Party A) rather than the political elite (politicians) as the source of political contamination. Insofar as political animosity is driven more by attitudes to out-group politicians than by attitudes toward supporters of an out-group party (Kingzette, 2021), using party norms provides a more conservative test of our hypotheses.

Furthermore, Study 2 tested the out-group- and in-group-effects both when there was a positive party-product association (e.g., Party A loves Product X) and when there was a negative party-product association (e.g., Party A hates Product Y), whereas Study 1 only tested the positive association. It also investigated how a bipartisan political association (e.g., both the most- and least-liked parties love Product X) influenced subsequent evaluations. Establishing whether an in-group association trumps an out-group association or vice versa with the same product is important theoretically, as it tells us something about the relative strength of positive and negative political identities.

Finally, Study 2 used a more controlled manipulation in that the same products were given an out-group and/or in-group association. Whereas the least- and most-liked clothes varied as a function of each participant’s least and most-liked party in Study 1, participants in Study 2 were randomly assigned to conditions that determined which type of political association (least-liked, most-liked, bipartisan, or none) that was given to relatively popular and unpopular chocolates.

### Method

We requested 800 complete responses, and a sensitivity power analysis revealed that 200 participants in each condition would yield 80% power to detect an effect size of *d* = .18 in paired *t* tests of changes in evaluations of chocolates. The total sample size was 813. The participants were informed that the purpose of the study was to investigate people’s attitudes toward chocolate bars. They were randomly assigned into four conditions: (a) least-liked party (*n* = 205), (b) most-liked party (*n* = 195), (c) bipartisan (*n* = 209), and (d) control (*n* = 204).

First, all participants provided their initial Time 1 ratings of eight chocolate bars that are well-known in Sweden (Geisha, Snickers, Twix, Dumle, Daim, Marabou, Riesen, and Fazermint) presented on the same page on visual analog scales ranging from −50 (“Dislike extremely much”) to +50 (“Like extremely much”). On the following page, participants responded to demographic questions (see Table 1). They were thereafter asked to choose their least-liked party and/or their most-liked party among the eight parties in the Swedish parliament.

Next, all participants provided Time 2 ratings of chocolates, which were now presented on separate pages. The first four pages presented three *novel chocolates* (Plopp, Mars, and Toblerone) that had not been rated at Time 1, plus an embedded attention check. These were followed by three “*neutral chocolates*” (Geisha, Snickers, and Twix), which were not associated with any political party at Time 2, and thereafter three “*popular chocolates*” (Dumle, Daim, and Marabou) and two “*unpopular chocolates*” (Riesen and Fazermint). Classification of these chocolates was based on a pilot study with a representative sample of 733 Swedes that showed that the “popular” and “unpopular” chocolates were among the most and least popular, respectively, among supporters of *all* parties.

**Figure 2.**
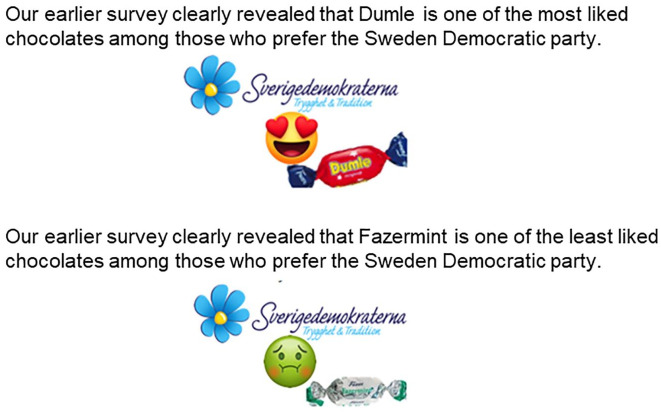
Example of Political Associations With a Popular and an Unpopular Chocolate (Translated From Swedish).

Participants in the control condition rated all chocolates at Time 2 without any additional information. Participants in the three experimental conditions received information about the abovementioned pilot-study prior to rating the chocolates at Time 2. Also, when evaluating each popular chocolate, participants in the experimental conditions learned that the pilot-study revealed that this chocolate was among the *most preferred by supporters of* (1) “[name of least-liked party]” (least-liked condition), (2) “[name of most-liked party]” (most-liked condition), or (3) “[names of most-liked and least-liked parties]” (bipartisan condition). This illustrates a *positive* party-to-product association. Similarly, when evaluating each unpopular chocolate, they learned that the pilot-study revealed that this chocolate was among the *least preferred* by supporters of their least-liked or most-liked party, or both, which illustrates a *negative* party-to-product association. The associations were communicated both in text and with a graphical illustration including the party symbol, an emoji (the heart-eyes emoji for the positive association and the nauseated green face emoji for the negative association), and a picture of the chocolate (see Figure 2). No information about partisan preferences was given when the novel and neutral chocolates were evaluated.

After the Time 2 ratings of all chocolates, all participants chose their most-liked chocolate among the eight included chocolates (see Supplementary Material). We then measured affective polarization using either the feeling thermometer or the social distance measure (participants were randomized to one of the measures). Finally, all participants completed a brief measure of bullshit receptivity (not reported).

### Results

Following the preregistration, we created four chocolate-type variables (aggregated ratings of the novel, neutral, popular, and unpopular chocolates). Preliminary tests confirmed that there were no significant differences between conditions in ratings at Time 1: *F*(3,809) = 0.39, *p* = .76, η_p_^2^ <.01 for neutral chocolates; *F*(3,809) = 0.55, *p* = .65, η_p_^2^ <.01 for popular chocolates; and *F*(3,809) = 0.47, *p* = .70, η_p_^2^ <.01 for unpopular chocolates. Similarly, there were no significant differences between conditions in ratings of the novel chocolates at Time 2, *F*(3,809) = 1.01, *p* = .39, η_p_^2^ <.01.

An initial 3(Chocolate type: neutral/popular/unpopular) × 4(Condition: least-liked party/most-liked party/bipartisan/control) × 2(Time: 1[before political association]/2[after political association]) × 2(Participant partisanship: leftist/rightist) mixed ANOVA revealed the expected Chocolate type × Condition × Time three-way interaction, *F*(6, 1610) = 4.59, *p* < .001, η_p_^2^ =.02, which shows that the effect of the experimental manipulation differed across types of chocolates. The four-way interaction provided no evidence that leftists and rightists were differently impacted by the manipulation, *F*(6, 1610) = 0.65, *p* = .692, η_p_^2^ <.01.

**Figure 3.**
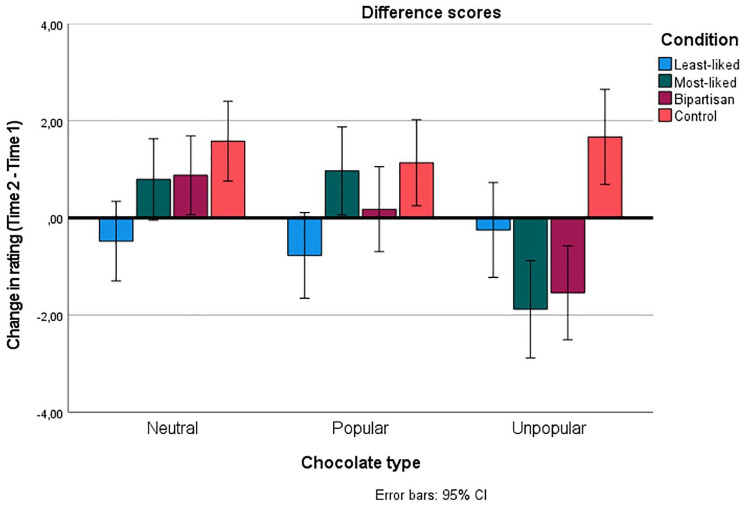
Change in Ratings of Each Chocolate-Type as a Function of Condition. Values Over Zero Indicate a Positive Change (Higher Rating at Time 2) Whereas Values Below Zero Indicate a Negative Change (Lower Rating at Time 2). Error bars Illustrate 95% Confidence Intervals.

Notably, participants in the control condition consistently provided higher ratings of all chocolates at Time 2 (see Figure 3). These results can be explained in terms of initial attitudes becoming stronger following *repeated attitude expression* (Downing et al., 1992) and *repeated exposure* to the chocolates (Zajonc, 1968). We preregistered a comparison of changes in evaluations against zero (i.e., no change from Time 1 to Time 2), and paired t-tests comparing Time 1 and Time 2 evaluations for each chocolate-type in each condition are presented in Table 3. Nevertheless, it is arguably more suitable to compare the change in ratings in the three experimental conditions against the change in ratings in the control condition, as this controls for repeated exposure and expression (which is identical in all conditions). Consequently, we report independent t-tests which investigate how changes in the experimental conditions differed from changes in the control condition for each type of chocolate.

**Table 3.** Paired t-Tests Comparing Mean Evaluations of the Different Types of Chocolates at Time 1 (Before Partisan Association) and Time 2 (After Partisan Association). A Positive Effect Size Indicate That the Chocolate Type Became More Attractive at Time 2 Whereas a Negative Effects Size Indicate That It Became Less Attractive.

Chocolates	Condition			
	Least-liked	Most-liked	Bipartisan	Control
Neutral(No info)	*t*(204) = −1.07, *p* = .287, *d* = −.08	*t*(194) = 1.88, *p* = .061, *d* = .14	*t*(208) = 2.25, *p* = .025, *d* = .16	*t*(203) = 3.82, *p* <.001, *d* = .27
Popular (Liked by. . .)	*t*(204) = −1.69, *p* = .093, *d* = −.12	*t*(194) = 2.05, *p* = .041, *d* = .15	*t*(208) = 0.37, *p* = .712, *d* = .03	*t*(203) = 2.93, *p* = .004, *d* = .21
Unpopular (Disliked by. . .)	*t*(204) = −0.45, *p* = .655, *d* = −.03	*t*(194) = −4.29, *p* <.001, *d* = −.31	*t*(208) = −3.03, *p* = .003, *d* = −.21	*t*(203) = 3.44, *p* < .001, *d* = .24

#### Neutral Chocolates

The change in evaluations was more negative in the least-liked condition (*M*_change_ *=* −0.48, *SD* = 6.44) than the control condition (*M_change_ =* +1.58, *SD* = 5.92; *t*[407] = −3.37, *p* <.001, *d* = −.33), but the changes in the most-liked condition (*M_change_ =* +0.79, *SD* = 5.87) and the bipartisan condition (*M*_change_ *=* +0.88, *SD* = 5.64) did not differ from the change in the control condition (*t*[397] = −1.34, *p* = .181, *d* = −.13 and *t*[411] = −1.24, *p* = .217, *d* = −.12, respectively; see Figure 3). This, in combination with the absence of effect for novel chocolates at Time 2, indicates that political cues of one’s least-liked party can nullify the positive effect of repeated exposure and expressions.

#### Popular Chocolates

Participants in the most-liked party condition increased their ratings marginally at Time 2, whereas participants in the least-liked party condition showed a non-significant decrease in rating (see Table 3). These results are consistent with the hypothesized in-group- and out-group-effects, albeit not as clear as in Study 1. The change in ratings was more negative in the least-liked condition (*M_change_ =* −0.77, *SD* = 6.55) than in the control condition (*M_change_ =* +1.14, *SD* = 5.53; *t*[407] = −3.18, *p* = .002, *d* = −.32). In contrast, the most-liked condition (*M_change_ =* +0.97, *SD* = 6.58) and the bipartisan condition (*M_change_ =* +0.18, *SD* = 6.99) did not differ from the control condition (*t*[397] = −0.28, *p* = .782, *d* = −.03, and *t*[411] = −1.54, *p* = .124, *d* = −.15 respectively). These results provide partial support for the out-group hypothesis, but not the in-group hypothesis, when controlling for repeated exposure and expressions, and when the party-to-product association is positive.

#### Unpopular chocolates

Participants in the most-liked party condition and the bipartisan condition, but not in the least-liked condition, decreased their ratings of unpopular chocolates at Time 2. The changes in ratings in all experimental conditions were more negative than the changes in the control condition. However, there were medium-sized differences between the control condition (*M_change_ =* +1.67, *SD* = 6.92) and the most-liked (*M_change_ =* −1.88, *SD* = 6.12; *t*[397] = −5.41, *p* <.001, *d* = −.54) and bipartisan (*M_change_ =* −1.54, *SD* = 7.35; *t*[411] = −4.56, *p* <.001, *d* = -.45) conditions, while the difference between the control condition and the least-liked condition was small (*M_change_ =* −0.25, *SD* = 7.95; *t*[407] = −2.60, *p* =.010, *d* = −.26). This supports the in-group hypothesis, but not the out-group hypothesis when controlling for repeated exposure and expressions, and when the party to product association is negative.

### Summary of Study 2

The out-group- and in-group-hypotheses were mostly supported when the party-to-product association was positive, and the comparison was zero change between Time 1 and 2. These effects occurred even with the more conservative tests this study provided. There was also an in-group effect when the party-to-product association was negative (i.e., chocolates became less attractive when disliked by supporters of one’s most-liked party), but the out-group effect did not appear (i.e., chocolates did not become more attractive when disliked by supporters of one’s least-liked party). Study 2 also revealed that information about bipartisan liking or disliking of chocolates influenced people similarly (but less strongly) as information about in-group attitudes alone did.

Among participants in the control condition, all chocolates were consistently evaluated more positively at Time 2, and as mentioned, two explanations for these findings are mere exposure and repeated attitude expression. To control for these aspects, we compared changes in the experimental conditions against changes in the control condition. Analyzed this way, the results supported only the out-group effect when there was a positive association (chocolates liked by the political out-group lost attraction) and only the in-group effect when there was a negative association (chocolates disliked by the political in-group lost attraction).

## Study 3: Politically Contaminated Charities

We next aimed to investigate the consequences of the out-group and in-group effect—specifically, whether political distancing can influence real financial decisions. This tests the behavioral implications of distancing from politically contaminated neutral products (Baumeister et al., 2007). The behavioral outcome was measured through allocation tasks where participants were asked to decide which of two charitable organizations to allocate donations to.

Unlike earlier studies, Study 3 used a between-group experimental design to test the out-group and in-group effects. As in Study 2, participants were randomly assigned to one of four conditions, which differed only in what kind of descriptive norm information participants were provided with. Participants in the least-liked [most-liked] condition were informed that supporters of their political out-group [in-group] preferred organization A over organization B, whereas participants in the bipartisan condition were informed that supporters of both their out-group and in-group did so. Participants in the control condition allocated their donations without any information about descriptive norms.

### Method

We requested 1,200 complete responses. A sensitivity power analysis revealed that 300 participants in each condition would yield 80% power to detect an effect size of *d* = .20 in independent t-tests. The total sample size was 1,239. Participants were told that the purpose of the study was to investigate people’s preferences regarding charitable giving. They were randomly assigned into four conditions: (a) least-liked party (*n* = 299), (b) most-liked party (*n* = 298), (c) bipartisan (*n* = 315), and (d) control (*n* = 327).

First, participants responded to demographic questions (see Table 1). Participants in the three experimental conditions also chose their least-liked party and/or their most-liked party. Next, participants were informed that they would be presented with seven tasks in which their job was to decide which out of two charity organizations would receive 1 SEK (≈ $0.12). They were assured that their decision would have real-life consequences (the first author later donated 8673 SEK in line with their decisions).

The allocation tasks were designed so that one organization was more popular than the other. The relative popularity of organizations was determined based on the results of a pilot-study (the same as the one used for Study 2) where participants reported their most-liked political party and expressed their attitudes and behavioral intentions toward 15 of the largest and most well-known charity organizations in Sweden. Although supporters of different parties varied in their attitudes toward charitable organizations, the organization defined as “popular” in our allocation tasks was, with few exceptions, evaluated more positively than the contrasting organization among supporters of *all* political parties. Table 4 shows the organizations that were pitted against each other in the order in which they presented to participants. The order of the organizations within each allocation task was randomized. The first four tasks pit organizations that focus on similar causes (e.g., international aid, medical research, or environmental work) against each other. To increase the number of observations, we also included three tasks where the organizations focused on different causes. One attention check was embedded among the allocation tasks.

**Table 4:** Proportion of Participants Who Chose the Popular Organization (in Bold Font) in Each Allocation Task by Condition.

Allocation tasks	Least-liked	Most-liked	Bipartisan	Control	χ^2^ (df =3)	*p*	*Cramer’s V*
**(1) Doctors without borders** vs. UNICEF	51.8%	59.1%	61.3%	62.7%	8.83	.032	.084
**(2) The Swedish Cancer Society** vs. the Swedish Brain Foundation	73.6%	74.5%	76.8%	74.3%	0.98	.807	.028
**(3) World Wildlife Foundation** vs. The Swedish Society for Nature Conservation	59.5%	63.4%	59.7%	65.4%	3.39	.335	.052
(**4) Save the Children** vs. SOS children’s villages	60.5%	73.2%	72.4%	66.1%	14.65	.002	.109
**(5) The Swedish Childhood Cancer Fund** vs. The Swedish Red Cross	67.6%	72.5%	71.1%	70.3%	1.86	.603	.039
(**6) Swedish Sea Rescue Society** vs. Greenpeace	57.5%	64.4%	66.3%	68.2%	8.71	.033	.084
**(7) The Swedish Heart Lung Foundation** vs. Amnesty International	71.9%	75.2%	75.9%	76.8%	2.20	.533	.042

Before the allocation tasks, participants in the experimental conditions were informed that the results of the pilot-study revealed that some organizations were more popular than others. They also received information about party norms. For example, in allocation task 1, the participants in the least-liked condition read “Our study conducted last fall revealed that most supporters of [name of participant’s least-liked party] prefer to donate to Doctors without Borders rather than to UNICEF. Please choose which of these organizations you want to donate 1 SEK to.” Participants in the most-liked condition read the same text with the name of their most-liked party inserted instead. Participants in the bipartisan condition were informed that supporters of both their most-liked and their least-liked party preferred Doctors without Borders rather than UNICEF. Participants in the control condition chose an organization without any information about the pilot-study.

After the seven allocation tasks, all participants completed a feeling thermometer measure for each party and a preferred social distance measure (“how would you react if your child married a person from each party?”). Results from these variables are not reported here but are included in the openly available data files.

### Results

**Figure 4.**
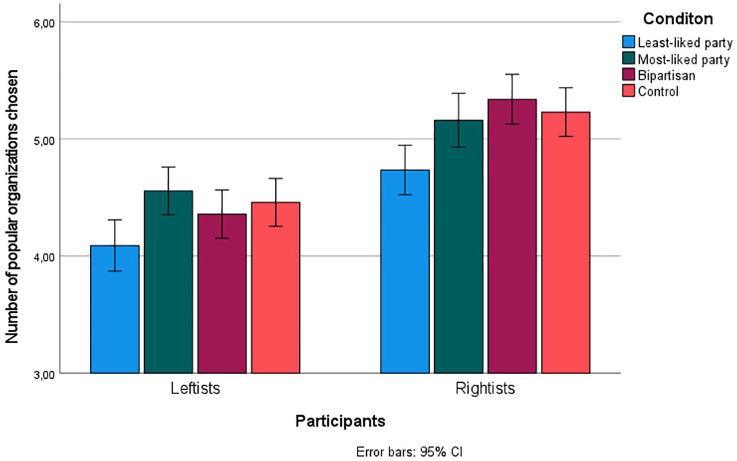
Mean number of Popular Organizations (Out of Seven) Chosen in Each Experimental Condition for Leftists and Rightists (Classified Based on Most-Liked Party). Error Bars Illustrate 95% Confidence Intervals.

A 4 (condition) × 2 (Participant partisanship: leftist/rightist) between groups ANOVA with the number of chosen “popular organizations” as the dependent variable, revealed a significant main effect on the condition, *F*(3, 1231) = 8.02, *p* <.001, η_p_^2^ = .02 (see Figure 4). Bonferroni-corrected post hoc tests revealed that people in the least-liked condition chose fewer popular organizations (*M* = 4.43, *SD* = 1.51) than people in the control condition (*M* = 4.84, *SD* = 1.33, *p* = .001, *d* = -.29), the most-liked condition (*M* = 4.82, *SD* = 1.33, *p* = .003, *d* = -.28), and the bipartisan condition (*M* = 4.83, *SD* = 1.41, *p* = .002, *d* = -.28). There were no differences between the other conditions (all *p’*s = 1). This supports the out-group hypothesis but not the in-group hypothesis.

There was also a significant main effect on participant ideology, *F*(1, 1231) = 96.37, *p* <.001, η_p_^2^ = .07, indicating that rightists chose more popular organizations overall. This is unsurprising considering that in all allocation tasks including organizations with different causes (Tasks 5–7), the “popular” organization focused primarily on helping in Sweden, whereas the “unpopular” organization focused on international causes, and studies have shown that rightists (vs. leftists) prefer domestic over international causes (Nilsson et al., 2020). The interaction effect was not significant, *F*(3, 1231) = 1.23, *p* = .296, η_p_^2^ < .01, meaning that there was no evidence of ideological asymmetries.

### Summary of Study 3

Study 3 replicated the out-group effect on real financial decisions in the prosocial domain. Participants allocated fewer donations to charities they learned were preferred by supporters of their least-liked party. However, there was no in-group effect in Study 3. As in Study 2, learning about bipartisan preferences influenced participants similar to learning about in-group preferences alone, and there was no evidence of differences in the strength of the negative out-group effect between leftists and rightists.

## Study 4: Indirect Political Distancing in Public and Private Situations

Finally, we tested if being observed by one’s in-group increases political distancing. If this is the case, it would indicate that social identity signaling influences attitudes and behavioral tendencies toward politically contaminated neutral products (Connors, 2023; Moore et al., 2023). As in Study 3, participants were presented with multiple product pairs. For each, they were asked to select which of the products they preferred. All products-pairs were again designed so that one product was more popular among supporters of *all* political parties (based on results from the abovementioned pilot-study). The dependent variable was once again how many “popular” products the participants chose.

### Method

We requested 1200 participants, and a sensitivity power analysis showed that this would give us 80% power to detect an effect size of *d* = 0.23 when making pairwise comparisons of conditions. The total sample size was 1295. Participants first reported demographic information, and most- and least-liked political parties (see Table 1). They also rated how strongly they identified as a supporter of their most-liked party and as an opponent of their least-liked party (on Likert-type scales from 0 = “*not at all*” to 10 = “*very strongly*”).

**Figure 5.**
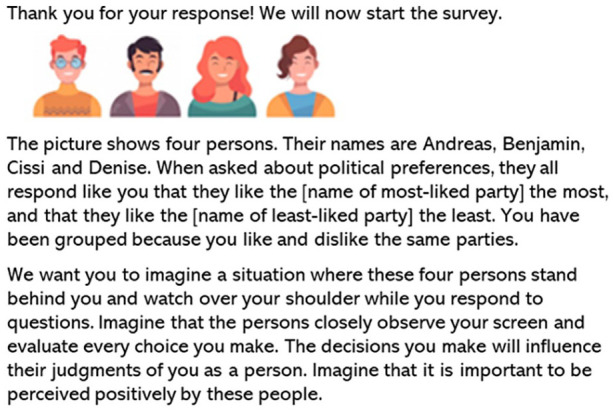
English Translation of the Introduction to the Four Observers (for Participants in the Public Conditions).

The participants were thereafter informed that they would choose the product (a charity, chocolate, or fruit) they “liked the most” in each of 20 product pairs (see Table 5). They were randomly assigned to five conditions: a control condition plus four experimental conditions in a 2(partisan association: least-liked party/most-liked party) × 2(decision setting: private/public) factorial design. Those in the control condition chose which product they preferred without additional information, whereas those in the experimental conditions were informed about the relative popularity of products among supporters of their most-liked or least-liked party. Moreover, prior to learning about the pilot-study, participants in the public conditions were introduced to four observers (see Figure 5). The observers were named and illustrated with animated faces, and it was made clear that they were paired with the participant because they had the same party preferences as the participant, and that they could evaluate the participant.

**Table 5.** Percentage of Participants in Each Condition Who Chose the Popular Product (in Bold) in Each Product Pair.

Product pairs	Conditions					
	Least-liked Public	Least-liked Private	Control	Most-liked Private	Most-liked Public	χ^2^ (*df* =4)
**World Wildlife Foundation** The Swedish Society for Nature Conservation	42.9%	50.6%	61.9%	73.0%	68.0%	64.2 *p* < .001
**Doctors without borders** UNICEF	48.0%	55.4%	67.3%	68.0%	70.6%	41.1 *p* < .001
**The Swedish Cancer Society** The Swedish Brain Foundation	59.9%	65.9%	82.9%	76.6%	78.8%	48.2 *p* < .001
**Save the Children** SOS children’s villages	49.6%	59.0%	67.6%	68.0%	74.0%	40.2 *p* < .001
**The Swedish Childhood Cancer Fund** The Swedish Red Cross	55.2%	63.1%	66.9%	72.5%	72.1%	23.4 *p* < .001
**Swedish Sea Rescue Society** Greenpeace	52.4%	58.2%	74.4%	70.5%	67.3%	37.6 *p* < .001
**Heart Lung Foundation** Amnesty International	62.3%	68.3%	69.0%	68.9%	77.7%	14.9 *p* = .005
**Save the Children** The Swedish Red Cross	46.8%	57.4%	62.3%	73.4%	75.5%	60.9 *p* < .001
**Doctors without borders** SOS children’s villages	50.4%	62.2%	73.0%	75.8%	72.5%	50.7 *p* < .001
**The Swedish Cancer Society** Heart Lung Foundation	61.9%	69.1%	79.7%	79.1%	80.3%	36.1 *p* < .001
**Daim** Snickers	45.6%	57.4%	63.0%	58.6%	57.2%	17.5 *p* < .001
**Marabou** Twix	59.5%	62.2%	61.6%	69.7%	64.7%	6.51 *p* = .164
**Dumle** Riesen	47.6%	53.8%	63.7%	57.4%	55.0%	14.7 *p* = .005
**Geisha** Fazermint	58.7%	63.9%	68.3%	68.9%	66.5%	7.7 *p* = .103
**Marabou** Riesen	54.4%	61.8%	62.6%	63.9%	62.1%	6.1 *p* = .194
**Tangerine** Orange	46.4%	55.8%	64.1%	61.5%	62.8%	22.4 *p* < .001
**Banana** Green Apple	57.5%	60.2%	67.6%	67.2%	66.5%	9.5 *p* = .049
**Grapes** Water melon	40.5%	36.5%	53.0%	50.8%	48.0%	20.1 *p* < .001
**Red Apple** Pear	43.3%	48.6%	55.2%	52.0%	54.6%	10.1 *p* = .039
**Orange** Green Apple	54.0%	59.0%	63.7%	62.7%	68.0%	12.3 *p* = .016

**Figure 6.**
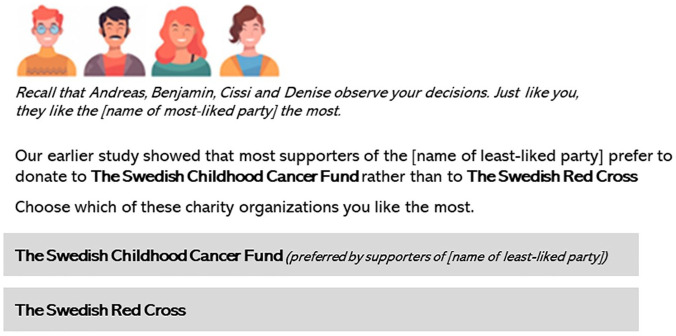
English Translation of an Example of a Product-Pair in the Least-Liked Party + Public Condition.

The product pairs were presented on separate pages in a fixed order (see Table 5). For each pair, participants in the least-liked [most-liked] party condition were reminded that the earlier study revealed that one product (the popular product) was generally preferred by supporters of the least-liked [most-liked] political party. On each of the 20 pages, participants in the public conditions were reminded about the four observers (see Figure 6).

After the final product-pair, all participants were asked to rate how observed they felt (on a scale from 1 = “not at all” to 5 = “very much”) when choosing products. They were thereafter asked to respond to 10 items from the need-to-belong scale (Leary et al., 2013).

### Results

**Figure 7.**
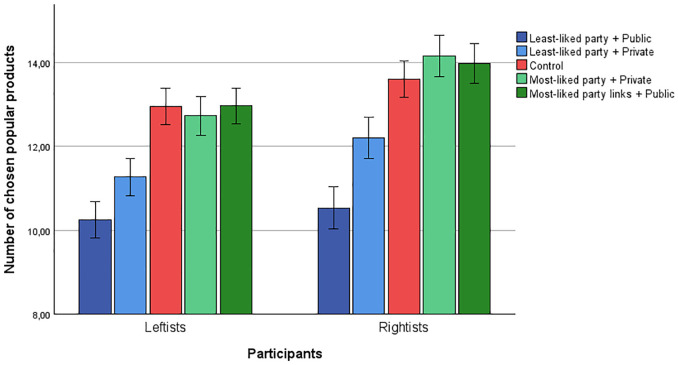
Average Number of Chosen Popular Products Among Leftists and Rightists (Based on Most-Liked Party) Divided by Condition. Error Bars Represent 95% Confidence Intervals.

A 5(condition) × 2(participant partisanship: leftist/rightist) between-group ANOVA with many chosen popular products as the dependent variable yielded a main effect of participant partisanship, *F*(4, 1285) = 33.49, *p* <.001, η^2^ = .025, meaning that rightist participants choose more popular products overall, as in Study 3. There was no interaction effect, *F*(4, 1285) = 1.59, *p* = .176, η^2^ = .005, to suggest that the experimental manipulation influenced leftists and rightists differently. More importantly, there was a main effect of condition *F*(4,1285) = 66.32, *p* <.001, η^2^ = .171 (see Figure 7).

A first planned contrast test showed that participants in the least-liked + private condition chose fewer popular products (*M* = 11.69, *SD* = 2.70) than participants in the control condition, *M* = 13.28, *SD* = 2.40; *t*(1290) = 6.80, *p* < .001, *d* = 0.59. This again replicates the negative out-group effect. A second planned contrast showed that participants in the most-liked + private condition chose about equally many popular products (*M* = 13.39, *SD* = 2.69) as participants in the control condition, *t*(1290) = 0.46, *p* = .647, *d* = 0.04. This means we did not observe any positive in-group effect.

We then investigated how decision setting influenced the two effects. A third planned contrast showed that participants in the least-liked + public condition chose fewer popular products (*M* = 10.37, *SD* = 3.00) than participants in the least-liked + private condition, *t*(1290) = 5.49, *p* <.001, *d* = 0.49. This indicates that the presence of in-group observers makes people even less likely to choose neutral products liked by their least-liked party. The fourth planned contrast showed that participants in the most-liked + public condition chose equally many popular products (*M* = 13.42, *SD* = 2.66) as participants in the most-liked + private condition, *t*(1290) = 0.16, *p* = .871, *d* = 0.01. This suggests that the presence of in-group observers does not make people choose more neutral products liked by their most-liked party.

Although directionally as expected, there were no significant condition differences in how observed participants felt when choosing products, *F*(4, 1290) = 1.11, *p* = .348. Possible reasons for this are the hypothetical nature of the task, and that a single item was used to measure feeling of being observed.

### Summary of Study 4

Study 4 again replicated the negative out-group in a private setting, but also provided experimental evidence that the tendency to avoid neutral products liked by supporters of the least-liked party gets stronger in public situations wherein people learn that others from the in-group observe their product choices. Like Study 3, there was no positive in-group effect in a private setting, and being observed did not make people choose more products liked by supporters of their most-liked party. These results indicate that social identity signaling is a contributing psychological mechanism of the negative out-group effect.

## General Discussion

This research contributes to the theoretical theme of cognitive consistency by demonstrating that people attitudinally distance themselves from neutral products that are preferred by leaders or supporters of their least-liked political party. In Study 1, participants found clothes less attractive after seeing a politician from their least-liked party wearing them (a negative out-group effect) and more attractive after seeing a politician from their most-liked party wearing them (a positive in-group effect). Similarly, participants in Study 2 evaluated chocolate bars that were loved by supporters of their least-liked party slightly less favorably and chocolates that were loved by supporters of their most-liked party slightly more favorably. Study 3 replicated the out-group effect (but not the in-group effect) on real financial decisions, showing that people allocated less money to charity organizations that were preferred among supporters of their least-liked party. Finally, Study 4 found that the negative out-group effect is stronger when people express product preferences in front of observers rather than in private. In sum, the results provided consistent evidence for the negative out-group effect, wherein products become less attractive when associated with the least-liked party, but only partial support for the positive in-group effect, wherein products become more attractive when associated with the most-liked party.

These four experiments are, as far as we know, the first to document this phenomenon. They show that political avoidance behaviors cut deeper into our everyday lives than previously known and extend well beyond situations where they can easily be seen as useful decision-making heuristics. This manifestation of affective polarization is different from political boycotting which implies avoiding brands owned by—or products sold by—explicit partisans (McConnell et al., 2018; Panagopoulos et al., 2020). Avoiding a restaurant whose owner is e.g., sponsoring the Trump campaign represents boycotting (and can perhaps be justified), whereas avoiding an apolitical restaurant where Trump once enjoyed a juicy burger represents indirect distancing (and is harder to justify as it can negatively affect the non-partisan restaurant owner).

### Unanswered Questions

The results from Study 2 were not as clear-cut as the results from the other studies. Possible reasons for this are the more complex design including also evaluations of products disliked by in-group or out-group partisans, the less identity-relevant products (chocolates), and the fact that the data collection happened to coincide with the Russian invasion of Ukraine in February 2022, which probably shifted the focus to Russia as the most prominent out-group while strengthening the sense of commonality and fellowship across party lines in Sweden. Moreover, when comparing changes in ratings in the experimental conditions against the changes seen in the control condition (to control for repeated exposure and attitude expression), Study 2 results indicated that partisan associations first and foremost make product evaluations more negative (in line with Klar et al., 2018). Specifically, the out-group effect emerged only when there was a *positive* party to product association (chocolates *liked* by the out-group are evaluated more negatively), whereas the in-group effect emerged only when there was a *negative* party to product association (chocolates *disliked* by the in-group are evaluated more negatively).

Across studies, the negative out-group effect is stronger and more consistent than the positive in-group effect. Speculatively, a contributing explanation for the absence of an in-group effect in Studies 3 and 4 could be that there is ambiguity in which decision is the most desirable when information about in-group preferences was provided. People might perceive the most normative outcome to be to allocate money to the non-preferred charity, because of a perceived preference for equity (Gordon-Hecker et al., 2017) or because of a greater perceived need for, or impact of, donations to the unpopular charity (Erlandsson et al., 2017). Another explanation is that the bad looms larger than the good, meaning that it is permissible and even expected to deviate from non-essential in-group norms (Konovalova & Le Mens, 2020), but that aligning with even one distinctive out-group norm creates cognitive inconsistency. Finally, people arguably have more opportunities to align with in-group norms which could diminish this effect in any given study.

Study 2 and Study 3 results suggest that learning about bipartisan liking of a product influences subsequent judgments and decisions similarly (albeit less strongly) as learning only about the most-liked party liking the product did. Similar findings for policy-evaluations were reported by Bolsen et al. (2014) and Cole et al. (2023). This suggests that the mere presence of an out-group is not sufficient to elicit distancing in product preferences, and that informing that in-group members also enjoy a product loved by the political out-group can neutralize or debias the negative out-group effect (e.g., not only Trump but also Obama and Taylor Swift enjoy cherry-vanilla ice cream).

### Differences Between Leftists and Rightists

There was no consistent evidence of ideological asymmetries. In Study 1, rightists but not leftist participants exhibited a positive in-group effect, whereas the negative out-group effect emerged in both groups but was slightly more pronounced among rightists. By contrast, there was no evidence of differences between leftists and rightists in political distancing in Studies 2 to 4. One explanation for this is that the source of contamination was party leaders in Study 1, but descriptive norms among the electorate in Studies 2 to 4, and it is possible that rightists like right-wing politicians more than leftists like left-wing politicians, but that the same does not apply to attitudes toward party supporters. Still, to determine whether this or something else (such as the type of product) explains diverging results across studies, controlled experiments dedicated to this are needed.

### Contributions, Limitations, and Future Directions

This research is rigorous in that the studies tested the out-group and the in-group effects on several types of products, employed different experimental paradigms, and used both party leaders and party supporters as the source of the political association. The fact that we found support for the out-group effect despite all these differences allows us to draw more general conclusions about political distancing. At the same time, our approach makes it harder to pinpoint for instance why the in-group effect emerged in some but not all studies.

While most previous research has used convenience samples from the United States, we recruited representative samples from Sweden, which has a multi-party system with an ideologically heterogeneous political landscape (Gidron et al., 2019). Unlike studies that investigate affective polarization in multiparty systems by contrasting only the two largest parties (e.g., Flores et al., 2022) or by contrasting people’s judgments of their most-liked party against judgments of all other parties on average (e.g., Renström et al., 2021), we operationalized the political in-group and out-group as one’s most-liked and least-liked parties (among the eight in the parliament). This least-liked party approach is important particularly insofar as out-group aversion is a stronger identity cue than in-group affection nowadays (Medeiros & Noël, 2014). Nevertheless, we recognize that also our approach is a simplification of reality and that it would be possible to compare not only the “primary” out-groups and in-groups against non-partisans but also “secondary” out- and in-groups (for instance, other parties in the same coalition as your least-liked and most-liked party). As there are several legitimate ways to classify participants in terms of party preference within a multiparty system, we repeated the analyses based on different classifications and found similar results (reported in the Supplementary Materials), providing further evidence of their robustness.

Across studies, it can be argued that political identity is salient when participants evaluate politically contaminated neutral products which could lead to experimental demand. Even if demand effects appear to be smaller in online studies with non-student participants (Coles & Frank, 2023), our participants might have been aware of what was being tested. However, when testing ethnicity- or gender-based prejudice and discrimination, people often compensate or even overcompensate when responding to explicit questions (e.g., Jørgensen et al., 2013), as an unequal valuation of people is typically seen as undesirable and hard to justify (Erlandsson et al., 2024). Likewise, research on political motivated reasoning indicates that people strive to see themselves as uninfluenced by partisan considerations (Pronin et al., 2002; Stanovich, 2021; Van Boven et al., 2018) and exaggerate the influence of political cues when making decisions for others (vs. themselves; Pomerance & Van Boven, 2025). This suggests that demand characteristics would reduce rather than increase partisan responses.

Study 4 provides initial insights into the conditions that produce the effects as the tendency to avoid neutral products liked by out-group partisans was stronger in a public situation, where participants imagined being observed by others from their in-group. The presence of an audience could boost the need for internal cognitive consistency (Baumeister & Tice, 1984), or people might anticipate that any expressed opinion that could be interpreted in term of an affinity with the political out-group will be socially sanctioned by the political in-group. This study should be seen only as a first step of testing underlying mechanisms of the effects. We acknowledge that our manipulation of observers as animated avatars is basic and somehow artificial and that future studies could use either confederates, or avatars ostensibly created by other participants as the observers. Moreover, the observers in Study 4 were explicitly in-group members meaning that they had the same most-liked and least-liked party as the participant. A natural next step would be to manipulate the identity of the individuals in the observing group. If the observed pattern remains when observers are neutral or from the out-group, this supports a public self-construction explanation where people are motivated to signal their social identity. If the effect disappears or becomes reversed when observers are from the out-group, this supports a strategic impression management explanation where people are motivated to express views that are congruent with the views of the present observers (Baumeister & Tice, 1984).

Other fruitful avenues for future studies could be to investigate how people perceive and behave toward co-partisans who openly adhere to out-group norms, and to test how political associations influence attitudes toward products or entities that are more important to people. Unlike one’s favorite chocolate, one’s favorite sports team can be highly identity-relevant (Rees et al., 2015). Speculatively, we predict an out-group effect among weak supporters of a sports team (i.e., decreased support if an out-group politician supported the team) but no effect or even a reversed effect among already devoted supporters (i.e., increased team-support to “decontaminate” the team from out-group-partisanship). Conversely, among individuals with a strong team-identity and a weak political identity, we might expect increased support or even voting for a party leader who supports the team.

## Conclusion

Previous research has shown that political distancing can be manifested directly by avoiding interactions with members of the political out-group, and indirectly by discarding political policies suggested by the out-group, or by boycotting brands supporting the out-group. The current research demonstrated indirect political distancing also when people evaluate completely neutral and apolitical products and provided insights into situational factors that moderate this effect. While other types of political distancing can in some cases be explained in terms of accuracy-based or justifiable motivations, the finding that people distance themselves even from completely apolitical products simply because these happen to be liked by leaders or supporters of a political out-group demonstrates the subtle impact affective political polarization has on our everyday behavior.

## Notes

1. See https://www.riksdagen.se/en/members-and-parties/the-parties-in-the-riksdag/; https://politpro.eu/en/sweden/parties.

2. In the spring of 2021, the Center and Liberal made up a liberal middle block, so participants who chose one of these parties as their most liked were not classified as leftist or rightist in Study 1. At the time of Studies 2 to 4, the Center Party had allied with the left block, whereas the Liberal party had allied with the right block.

3. The three dependent variables were transformed into a unison 0 to 8 scale prior to aggregating them.

4. The first additional picture was included to put the participants in a mind-set that they would evaluate new clothes in this round. The second additional picture served as an attention check.

5. We deviated from the preregistered plan in one way. We originally planned to aggregate evaluations of clothes worn by leftist and rightist politicians, respectively, and compare leftist and rightist participants’ changes in evaluations of all leftist and rightist clothes to increase the reliability of the estimates. We ultimately decided to report results from the analyses based on most- and least-liked party in the main text (described as secondary analyses in the pre-registration), because this classification more directly maps onto in-group and out-group affiliations, and there was substantial variability in the participants’ feelings toward different left- and right-wing parties. For example, some right-leaning participants strongly liked one right-leaning party while reporting only moderate liking or even dislike of other right-leaning parties. Results based on the preregistered classification of the clothes are reported in Supplementary Materials.
